# Replacement of Arg in the conserved N-terminal RLFDQxFG motif affects physico-chemical properties and chaperone-like activity of human small heat shock protein HspB8 (Hsp22)

**DOI:** 10.1371/journal.pone.0253432

**Published:** 2021-06-18

**Authors:** Vladislav M. Shatov, Nikolai N. Sluchanko, Nikolai B. Gusev

**Affiliations:** 1 Department of Biochemistry, School of Biology, Moscow State University, Moscow, Russian Federation; 2 A.N. Bach Institute of Biochemistry, Federal Research Center of Biotechnology, Russian Academy of Sciences, Moscow, Russian Federation; Russian Academy of Medical Sciences, RUSSIAN FEDERATION

## Abstract

The small heat shock protein (sHsp) called HspB8 (formerly, Hsp22) is one of the least typical sHsp members, whose oligomerization status remains debatable. Here we analyze the effect of mutations in a highly conservative sequence located in the N-terminal domain of human HspB8 on its physico-chemical properties and chaperone-like activity. According to size-exclusion chromatography coupled to multi-angle light scattering, the wild type (WT) HspB8 is present as dominating monomeric species (~24 kDa) and a small fraction of oligomers (~60 kDa). The R29A amino acid substitution leads to the predominant formation of 60-kDa oligomers, leaving only a small fraction of monomers. Deletion of the 28–32 pentapeptide (Δ mutant) results in the formation of minor quantities of dimers (~49 kDa) and large quantities of the 24-kDa monomers. Both the WT protein and its Δ mutant efficiently bind a hydrophobic probe bis-ANS and are relatively rapidly hydrolyzed by chymotrypsin, whereas the R29A mutant weakly binds bis-ANS and resists chymotrypsinolysis. In contrast to HspB8 WT and its Δ mutant, which are well phosphorylated by cAMP-dependent and ERK1 protein kinases, the R29A mutant is poorly phosphorylated. R29A mutation affects the chaperone-like activity of HspB8 measured *in vitro*. It is concluded that the irreplaceable Arg residue located in the only highly conservative motif in the N-terminal domain of all sHsp proteins affects the oligomeric structure and key properties of HspB8.

## Introduction

Small heat shock proteins (sHsp) form a large family of ubiquitously expressed chaperones detected in viruses, bacteria, plants and animals [1–3]. Monomers of sHsp have a low molecular mass varying between 13 and 43 kDa and contain a conservative α-crystallin domain (ACD) of 80–100 amino acid residues, which is usually located in the C-terminal part of the molecule [4, 5]. ACD is formed by seven or eight β-strands forming a compact β-sandwich, plays an important role in the intersubunit interaction and stabilizes the formation of sHsp dimers [6, 7]. ACD is flanked by variable and flexible N-terminal (NTD) and C-terminal (CTD) domains. Some sHsp contain in their C-terminal domain a conserved (I/V/L)-X-(I/V/L) tripeptide, the so-called anchoring motif, that can interact with a hydrophobic groove formed by β4-β8 strands of the neighboring dimer, thus providing for the interaction between two sHsp dimers [8, 9]. Protein partners (for instance, BAG3) of sHsp also contain in their structure the (I/V/L)-X-(I/V/L) tripeptides forming a docking site for sHsp recruitment [10, 11]. A similar hydrophobic tripeptide taking part in the interdimer interaction can alternatively be located within the NTD [12]. Besides, the NTD is implicated in the formation of very large homo- and heterooligomers of sHsp containing more than 20 subunits and participates in the interaction of sHsp with their client proteins [13–15].

The human genome contains ten genes encoding different members of the sHsp family [16, 17]. Among them, four members of this family, namely HspB1, HspB5, HspB6 and HspB8, are ubiquitously expressed in practically all human tissues [18]. While these proteins have similar primary structures, their quaternary structures are markedly different. For instance, HspB1 and HspB5 form large homo- or heterooligomeric complexes with an Mw of 400–600 kDa, whereas HspB6 and HspB8 form only small homooligomers and can be included in large heterooligomers only via interacting with HspB1 or HspB5 [15, 19–22]. Human sHsp contain in their NTD a remarkable (S/G)RL(F/L)D(Q/D)XFG sequence, where especially the arginine is absolutely conserved [23]. To investigate the role of this motif in the structure and properties of human sHsp, we replaced this conserved Arg residue by Ala or completely deleted the conserved (S/G)RL(F/L)D peptide (residues 28–32 of HspB8) [20, 21]. This paper deals with a detailed characterization and comparison of physico-chemical properties of the wild type HspB8 and its R29A and Δ28–32 mutants.

## Materials and methods

### Plasmid construction, protein expression and purification

The pET23b plasmid containing the human HspB8 sequence (referred to as HspB8 WT; Uniprot Q9UJY1), an HspB8 construct carrying the R29A mutation [20, 21] and the so-called Cys mutant of HspB8 (HspB8Cys) with the C10S/C99S/C195S/N138C amino acid substitutions [24, 25] were described earlier. To generate the Δ28–32 mutant, the cDNA of HspB8 WT was PCR-amplified using B8del_FW (5`-GACTCTCCCCTCTCCGATGGCTTTGGCATGGACC–3`) and B8del_Rev (5`-CATGCCAAAGCCATCGGAGAGGGGAGAGTCCCGG-3`) primers. The PCR product was digested by *Nde*I and *Xho*I restriction endonucleases and ligated into the pET23 plasmid. The integrity of all plasmids was verified by DNA sequencing (Evrogen, Moscow).

All constructs were transformed into *E*. *coli* BL21(DE3) cells, and single colonies were used to inoculate appropriate media. Expression of HspB8 WT and its Δ28–32 mutant was autoinduced by overnight incubation in 3-fold LB (Lysogeny broth) media as described earlier [21]. For expression of the R29A mutant, bacteria were grown on the standard LB media, and HspB8Cys was grown on Superior Broth (AthenaES) media at 37°C. After reaching an optical density at 600 nm of 0.6–0.8, expression was started by the addition of IPTG (the final concentration 0.5 mM) and lasted for 6 h. Bacterial cells were collected, suspended in lysis buffer (50 mM Tris-HCl pH 8.0, 150 mM NaCl, 0.1 mM EDTA, 0.1 mM phenylmethanesulfonyl fluoride (PMSF), 5 mM β-mercaptoethanol (ME)) and stored at -20°C.

Recombinant proteins were purified by using ammonium sulfate fractionation, hydrophobic-interaction and size-exclusion chromatography as described earlier [24, 26]. All samples were frozen and stored at -20°C in 20 mM Tris-acetate buffer pH 7.6, 10 mM NaCl, 0.1 mM EDTA, 0.1 mM PMSF and 2 mM dithiothreitol (DTT). The purity of proteins was no less than 95% according to SDS gel-electrophoresis [27]. Protein concentration was determined spectrophotometrically using A_280_^0.1%^ equal to 1.225 for HspB8 WT and its Cys and R29A mutants and equal to 1.260 for the Δ28–32 mutant of HspB8.

### Size-exclusion chromatography (SEC)

The quaternary structure was analyzed employing size-exclusion chromatography performed on a Superdex 200 HR 10/30 column equilibrated with buffer D containing 20 mM Tris/acetate (pH 7.6), 150 mM NaCl, 0.1 mM EDTA, 0.1 mM PMSF, and 15 mM ME. Samples containing 10–120 μg of protein dissolved in 100 μL of buffer were loaded on the column and eluted with the rate of 0.5 ml/min. The column was calibrated with thyroglobulin (660 kDa), ferritin (440 kDa), aldolase (158 kDa), conalbumin (75 kDa), ovalbumin (43 kDa), carboanhydrase (29 kDa) and RNAse (13.7 kDa).

Size-exclusion chromatography with multi-angle light scattering (SEC-MALS) was performed on a Superdex 200 Increase 10/30 column (GE Healthcare) coupled to a UV-Vis Prostar 335 detector (Varian) and a miniDAWN detector (Wyatt Technology). For SEC-MALS, 200–250 μg of either HspB8 WT or R29A, or 400 μg of Δ28–32 were applied to the column equilibrated with buffer D that contained 2 mM DTT instead of ME, or 200–250 μg of oxidized HspB8Cys was applied in buffer D without any reducing agents. Signals from the detectors were processed with ASTRA 8.0 software (Wyatt Technology) using protein-specific extinction coefficients indicated above and dn/dc equal to 0.185. Oxidation of the Cys mutant of HspB8 was achieved by overnight dialysis against 50 mM Tris-HCl buffer, pH 8.0, 50 mM NaCl, 1 mM MgCl_2_ at +4°C [25]. Completeness of disulfide crosslinking was checked by SDS-PAGE in the absence of ME and was no less than 90%.

### Fluorescence spectroscopy

All experiments were performed on a CaryEclipse (Varian) spectrofluorometer in a temperature-controlled cell (30°C) in phosphate buffer (50 mM KH_2_PO_4_, pH 7.5, 150 mM NaCl, 0.1 mM EDTA, 15 mM ME). Recombinant proteins were titrated by fluorescent probe bis-ANS (Molecular Probes). Protein samples (0.05 mg/ml, or 2.3 μM per HspB8 monomer) were titrated by a stock solution of bis-ANS, so that the final concentration of the probe was in the range of 1–15 μM. Fluorescence was excited either at 295 (to excite Trp and bis-ANS) or 385 (to excite only bis-ANS) nm (slit width 5 nm) and emission spectra were recorded in the range of 305–590 nm or 400–590 nm (slit width 5 nm).

### Limited chymotrypsinolysis

Isolated HspB8 WT and its R29A and Δ28–32 mutants (0.5 mg/ml, or 14 μM per monomer) were subjected to proteolysis in 20 mM Tris/acetate buffer pH 7.6, 10 mM NaCl, 0.1 mM EDTA, 15 mM ME. The reaction was started by the addition of TLCK-treated chymotrypsin (Sigma) so that the substrate protein/chymotrypsin weight ratio was kept constant at 7000: 1. The samples were incubated at 37°C for 5, 15, 30, 60 and 100 min. The reaction was stopped by the addition of PMSF up to 1 mM and subjected to SDS-PAGE on 15% polyacrylamide gels [27].

### Phosphorylation

ERK1 was activated by incubation with constitutively active MEK (mitogen activated protein kinase kinase) for 1 h at 37°C in 25 mM HEPES/KOH buffer pH 7.5, 25 mM phosphoglycerate, 4 mM MgCl_2_, 2 mM DTT, and 0.6 mM ATP. Activated ERK1 or the catalytic subunit of cAMP-dependent protein kinase were added to the incubation mixture containing different HspB8 species (0.5 mg/ml) and incubated for a different time at 37°C. Aliquots of the incubation mixture were subjected to urea PAGE [28, 29].

### Chaperone-like activity assay

Chaperone-like activity was determined by the ability of HspB8 species to prevent aggregation of partially denatured proteins [26]. To assess the chaperone-like activity of HspB8 and its mutants, porcine insulin and yeast alcohol dehydrogenase were used as model protein substrates. Aggregation of insulin was induced by the reduction of disulfide bonds [26, 30]. Insulin (5–7 mg/ml) was first dissolved in 2.5% acetic acid, incubated at +4°C overnight and centrifuged (12,000 g, 20 min, +4°C). The pellet was discarded and the supernatant was used for the aggregation assay. HspB8 (0.26 mg/ml) was dissolved in phosphate buffer (50 mM KH_2_PO_4_, pH 8.1, 100 mM NaCl, 20 mM DTT) and pre-incubated for 5 min at 37°C. Afterward, 10 μl of stock insulin solution was added to the incubation mixture (290 μl) containing HspB8. The final pH of the incubation mixture and the insulin concentration were equal to 7.2 and 0.2 mg/ml, respectively. Insulin aggregation was followed by measuring optical density at 360 nm on an Ultrospec 3100 Pro spectrophotometer (Amersham Pharmacia).

Yeast alcohol dehydrogenase (yADH) (0.16 mg/ml) was dissolved in phosphate-buffered saline, mixed with HspB8 species (0.32 mg/ml) in 280 μl and pre-incubated for 10 min at room temperature. Thus obtained mixture was transferred to a spectrophotometric cell and heated up to 42°C. Aggregation was induced by the addition of 20 μl of 300 mM DTT and aggregation of denatured yADH was followed by measuring optical density at 360 nm using an Ultrospec 3100 Pro spectrophotometer.

## Results

### Quaternary structure of HspB8 and its mutants analyzed by size-exclusion chromatography

In good agreement with the earlier published data [20, 26, 31], we found that on size-exclusion chromatography (SEC) HspB8 WT elutes as an entity with an apparent Mw of 33–36 kDa. The deletion mutant of HspB8 had a similar apparent Mw, whereas the apparent Mw of the R29A mutant increased up to ~60 kDa [20]. The Mw of human HspB8 calculated from its sequence is equal to 21604 Da. Therefore, the apparent Mw values derived from column calibration using protein standards cannot provide an accurate information on the quaternary structure of HspB8 and its mutants in solution, especially given the pronounced intrinsically disordered character of HspB8 [32].

**Fig 1:**
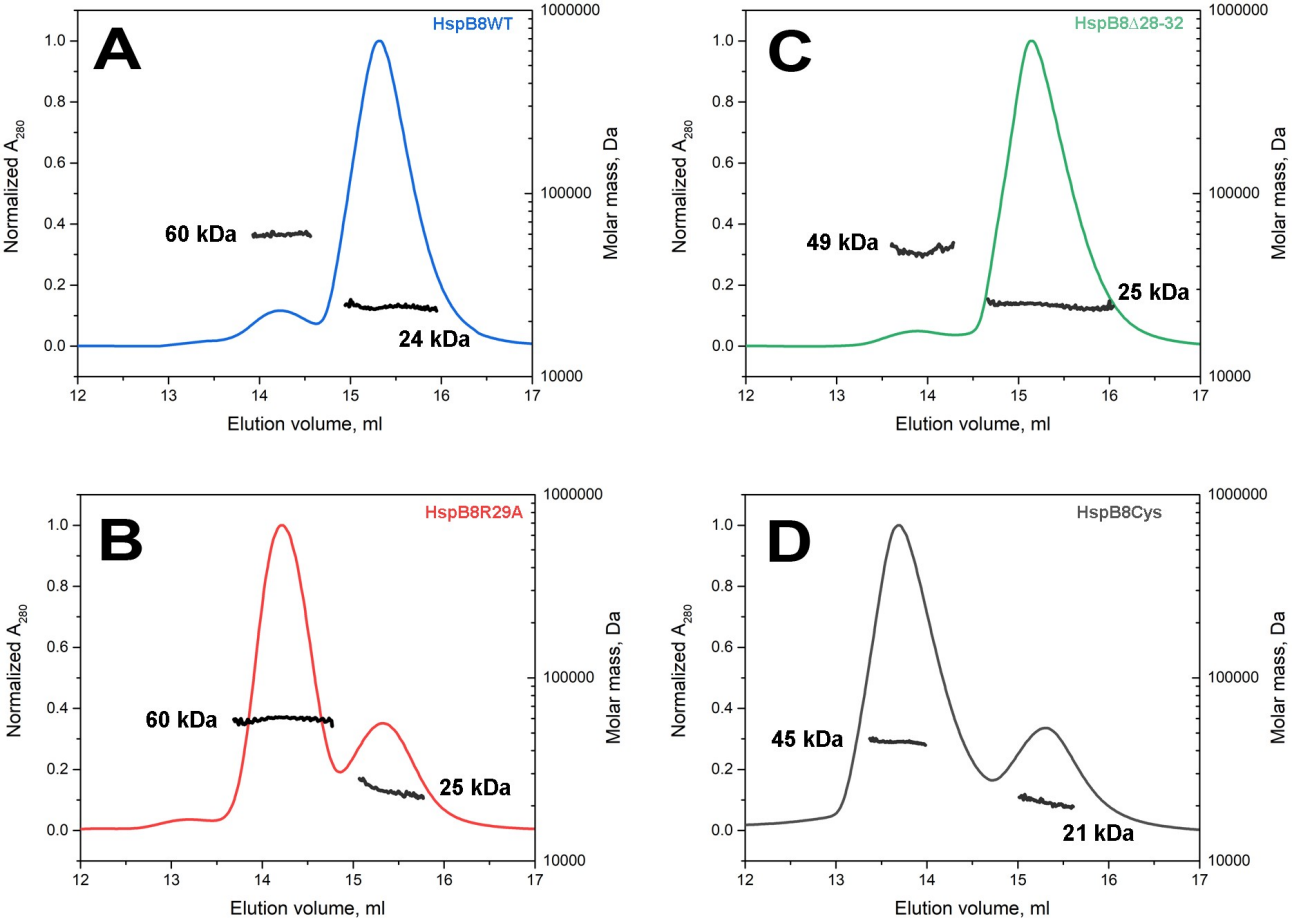
SEC-MALS of HspB8 WT (А), (blue), R29A (B), (red), Δ28–32 mutant (C), (green) and oxidized Cys mutant (D), (black). Chromatography of HspB8 WT, R29A and Δ28–32 mutants was performed in the presence of 2 mM DTT, whereas the oxidized Cys mutant was run without reducing agents. Numbers represent Mw (in kDa) averaged across each peak of different HspB8 species as determined by MALS.

To make a more accurate assessment, we used a combination of SEC with multi-angle light scattering (MALS) (Fig 1). When 200–250 μg of HspB8 WT were loaded on the Superdex 200 column, two peaks on the elution profile were detected. A smaller peak contained proteins with a MALS-derived Mw of 58–60 kDa, whereas the larger peak had a MALS-derived Mw of 23–24 kDa that was very close to the expected Mw of the HspB8 monomer (21.6 kDa). The polydispersity (the M_w_/M_n_ ratio, i.e. ratio of weight-averaged and number-averaged molecular masses, respectively) of both peaks was in the range of 1.001–1.003, thus indicating that highly homogeneous particles are contained in both peaks (Table 1). The R29A mutant also eluted as two peaks. In this case, however, the first, larger peak corresponded to Mw of 57–60 kDa, while the second, much smaller peak contained proteins with Mw of 24–25 kDa (Fig 1). Again, both peaks were represented by highly monodisperse particles (Table 1). The Δ28–32 mutant of HspB8 was eluted as two peaks: a very small peak with Mw of 48 kDa and a large peak with Mw of 23–25 kDa (Fig 1). In the control experiment, we subjected to SEC-MALS the oxidized Cys-mutant of HspB8 (Fig 1), which harbors an engineered disulfide bridge fixing the ACD in the dimeric state [24]. Although this protein again eluted as two peaks, their Mw values were close to 45–48 and 23–25 kDa, almost perfectly matching dimeric and monomeric species, respectively. It is worthwhile mentioning that the high Mw peak of the Δ28–32 mutant or that of the oxidized Cys mutant (45–48 kDa) had an elution volume smaller than that of the high Mw peak of the R29A mutant (57–60 kDa) (~13.7 vs ~14.2 ml, see Fig 1). This means that the R29A oligomers are significantly more compact than dimers formed by the oxidized Cys mutant or the Δ28–32 mutant of HspB8.

**Table 1:** Molecular masses of different HspB8 species.

Protein	Calculated Mw of monomer (kDa)	Apparent Mw derived from column calibration (kDa)	Mw and polydispersity derived from SEC-MALS			
			Peak 1		Peak 2	
			Mw (kDa)	Polydispersity	Mw (kDa)	Polydispersity
HspB8 WT	21.6	33–36	59.0 kDa (±1.405%)	1.000 (±1.99%)	25.3 kDa (±1.220%)	1.000 (±1.73%)
HspB8 R29A	21.5	59–62	59.8 kDa (±0.302%)	1.000 (±0.42%)	24.2 kDa (±1.009%)	1.004 (±1.42%)
HspB8 Δ28–32	21.0	33–38	48.6 kDa (±2.601%)	1.002 (±3.69%)	24.6 kDa (±0.619%)	1.001 (±0.88%)
HspB8Cys	21.5	38–39 (Red)* 79–82 (Ox)**	44.0 kDa (±0.439%)	1.001 (±0.62%)	20.9 kDa (±2.212%)	1.002 (±3.12%)

*—Apparent Mw of the main peak of reduced HspB8Cys

**—Apparent Mw of the main peak of oxidized HspB8Cys

These data indicate that while both HspB8 WT and its R29A mutant form monomers (23–24 kDa) and compact oligomers (57–60 kDa), their prevailing forms are opposing: HspB8 WT predominantly forms monomers, whereas the R29A mutant mainly forms compact oligomers. We assume that the equilibrium between these forms is established rather slowly because the two separate peaks are detected on the elution profiles at an approximately constant ratio, almost unchanged with load protein concentration. Neither the Δ28–32 mutant nor the oxidized Cys mutant formed compact oligomers with Mw of 57–60 kDa. Dimers formed by these proteins are less compact and have Mw of 45–48 kDa.

**Fig 2:**
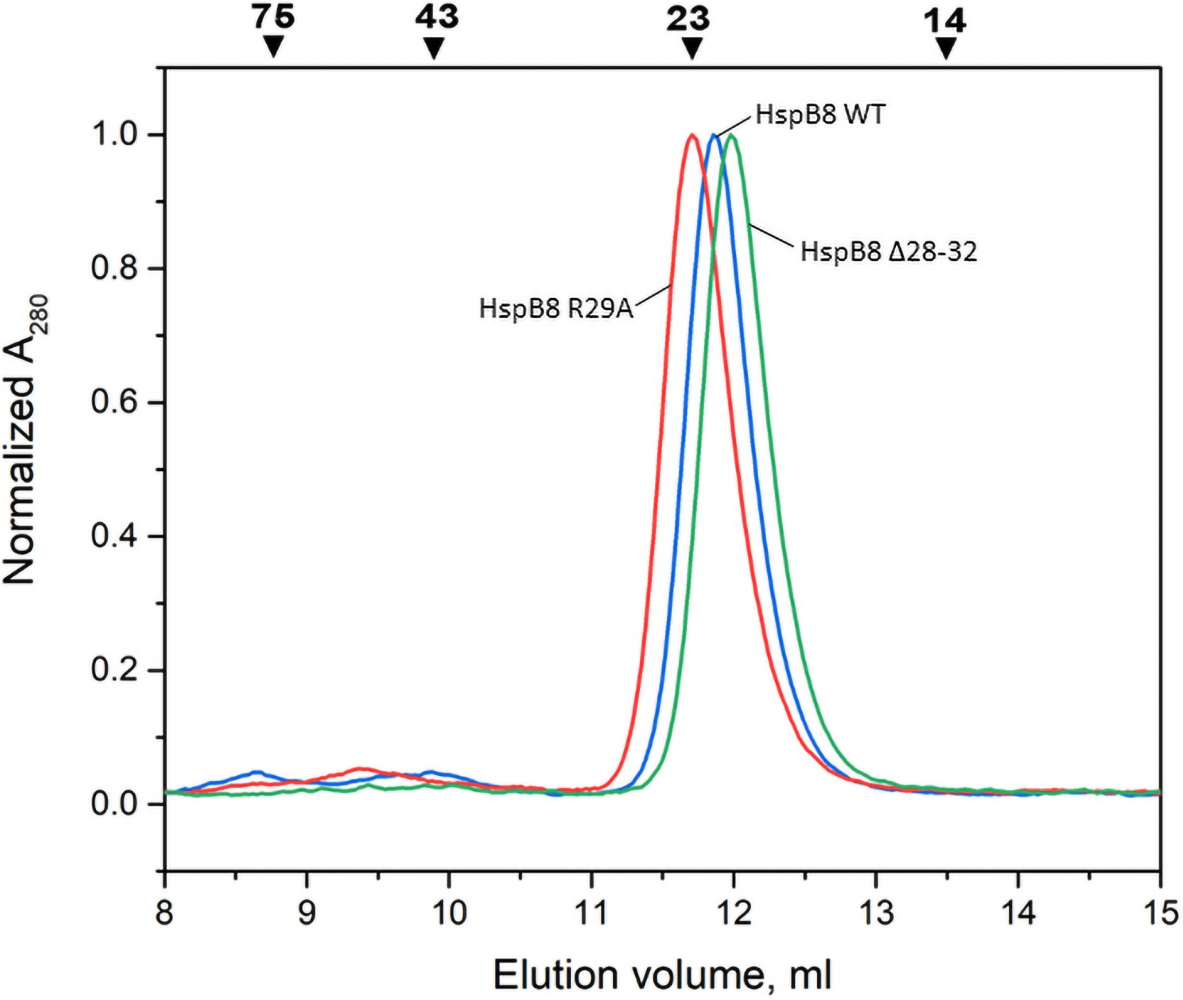
Size-exclusion chromatography of HspB8 WT (blue), R29A (red) and Δ28–32 mutant (green) in the presence of 6 M guanidinium chloride. Apparent Mw values (kDa) are indicated by arrows.

To confirm that all high Mw species detected on SEC are presented by differently assembled HspB8 subunits, we performed SEC in the presence of 6 M guanidinium chloride. As seen from Fig 2, all three, HspB8 WT and its R29A and Δ28–32 mutants, eluted as single peaks with apparent Mw values of 22–24 kDa indicating monomeric species.

### Spectral properties of HspB8 WT and its mutants

**Fig 3:**
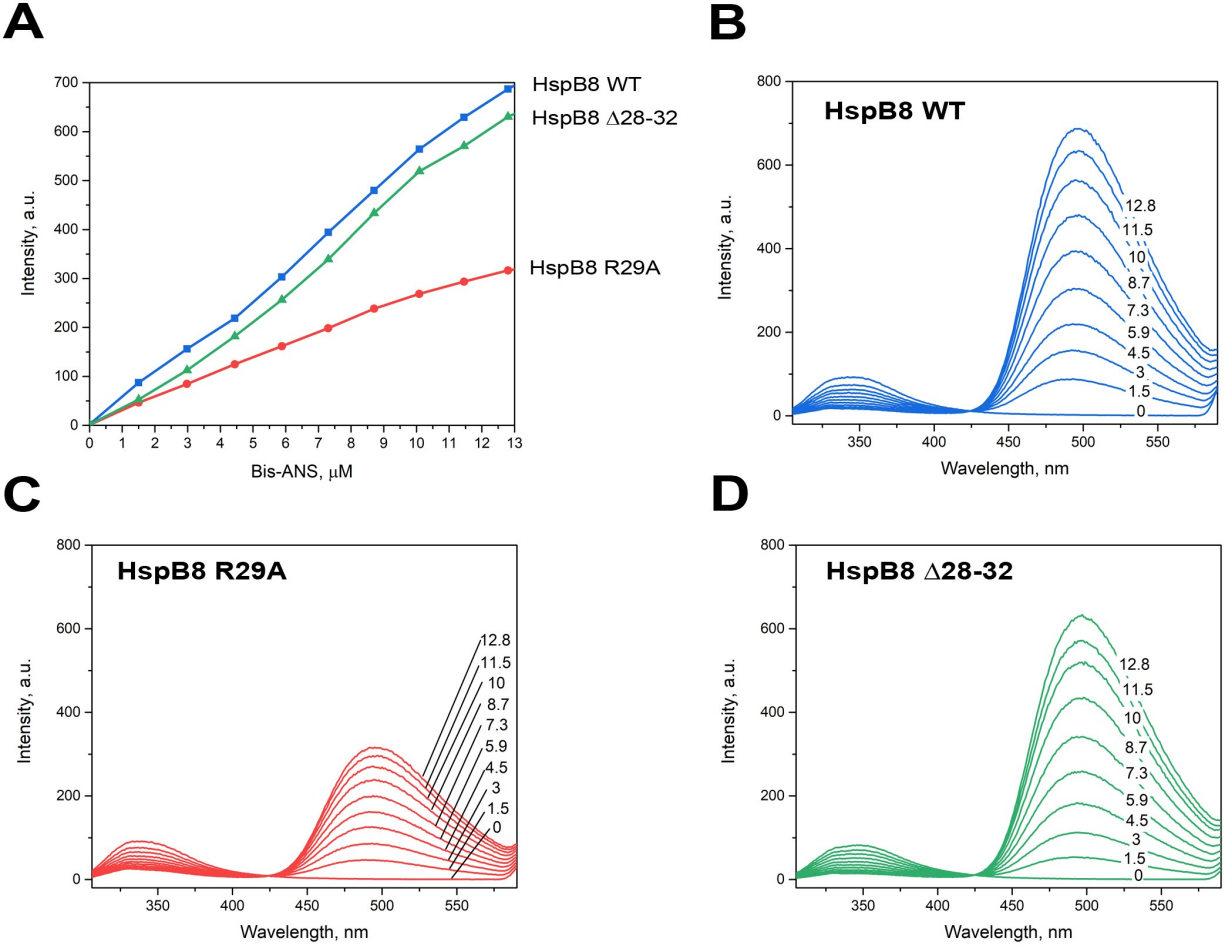
Titration of HspB8 WT and its mutants with hydrophobic probe bis-ANS. A. Increase of bis-ANS fluorescence excited at 385 nm and recorded at 495 nm for HspB8 WT (blue), R29A (red) or Δ28–32 (green) mutants. B, C, D. Fluorescence spectra excited at 295 nm and recorded after the addition of various bis-ANS concentrations (numbers in μM on panels) of bis-ANS to 2.3 μM of either HspB8 WT (panel B), the R29A or the Δ28–32 mutant (panels C and D), respectively.

Exposed hydrophobic surface of HspB8 and its mutants were analyzed by using the hydrophobic probe bis-ANS. Titration of HspB8 WT and its Δ28–32 mutant by bis-ANS was accompanied by a significant, almost linear increase of fluorescence at 495 nm excited at 385 nm (Fig 3A). By contrast, titration of the R29A mutant was accompanied by a much smaller increase of bis-ANS fluorescence (Fig 3A), thus indicating that this mutant is less hydrophobic than its WT counterpart. Similar results were obtained when bis-ANS fluorescence was excited at 295 nm, i.e. at the maximum of Trp absorbance (Fig 3B–3D). The Trp emission maximum of HspB8 is located at around 340 nm, indicating that Trp residues of HspB8 are predominantly solvent-exposed [32]. Although the difference between peaks of Trp emission and the probe excitation is rather large, Trp excitation induces bis-ANS fluorescence and the addition of bis-ANS induced quenching of Trp fluorescence. Quenching of Trp fluorescence induced by bis-ANS was less efficient in the case of R29A mutant than in the case of HspB8 WT or its Δ28–32 mutant. At the same time, bis-ANS fluorescence intensity at 495 nm was higher in the case of HspB8 WT and its Δ28–32 mutant than in the case of the R29A mutant (Fig 3B–3D). These data indicate that the R29A mutation affects energy migration from Trp to bis-ANS and the hydrophobic properties of HspB8.

### Effect of mutations in the N-terminal domain on limited chymotrypsinolysis of HspB8

**Fig 4:**
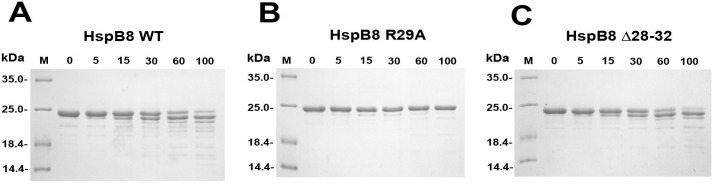
Limited chymotrypsinolysis of HspB8 WT (A), and its R29A (B) and Δ28–32 (C) mutants. Incubation time (min) is indicated above each lane. Positions of protein markers with their Mw values (in kDa) are marked by lines.

Limited proteolysis was used for further analysis of the effects of the R29A and Δ28–32 mutations on the structure of HspB8. As seen from Fig 4, incubation of HspB8 WT or its Δ28–32 mutant with chymotrypsin was accompanied by a relatively rapid degradation of the intact protein and accumulation of its proteolytic fragments. Under identical conditions, the R29A mutant resisted proteolysis and was not significantly cleaved even after 100 min incubation.

### Phosphorylation of HspB8 and its mutants by cAMP-dependent and ERK1 protein kinases

**Fig 5:**
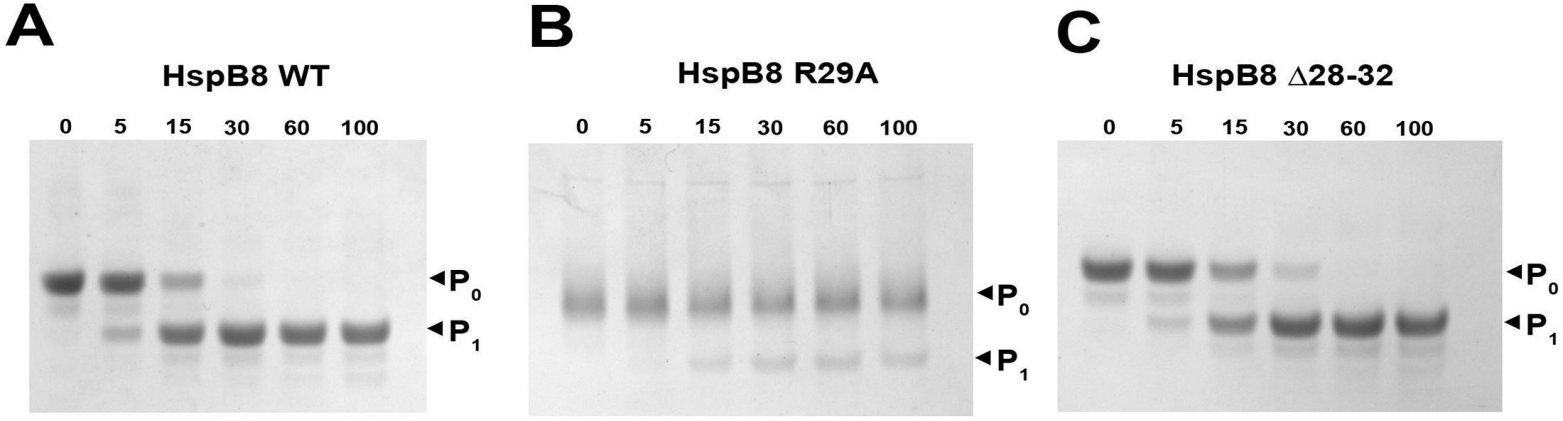
Phosphorylation of HspB8 and its mutants by cAMP-dependent protein kinase. Urea gel-electrophoresis of HspB8 WT (A), and its R29A (B) and Δ28–32 (C) mutants in the course of phosphorylation. Incubation time (min) is indicated above each lane. P_0_ represents a band of protein with no phosphate groups, P_1_ represents a band of protein with one phosphate group.

All earlier described sites of HspB8 phosphorylation (Ser14, Ser24, Ser27, Ser57, Thr63, Thr87) are located in the NTD [23]. Therefore, it was reasonable to analyze the effect of mutations in the NTD on HspB8 phosphorylation. The data of Fig 5 indicate that HspB8 WT and its Δ28–32 mutant were both efficiently phosphorylated by the catalytic subunit of cAMP-dependent protein kinase, whereas phosphorylation of the R29A mutant was negligibly low. Phosphorylation was accompanied by an increase of electrophoretic mobility of HspB8 on urea gel-electrophoresis. Incubation of HspB8 WT or its Δ28–32 mutant with cAMP-dependent protein kinase for as little as 30 min was accompanied by a complete disappearance of the band corresponding to the unphosphorylated protein and an appearance of an intense band corresponding to the monophosphorylated protein (Fig 5A and 5C). On the contrary, even the prolonged incubation of the R29A mutant with the protein kinase had no significant effect on its electrophoretic mobility (Fig 5B).

**Fig 6:**
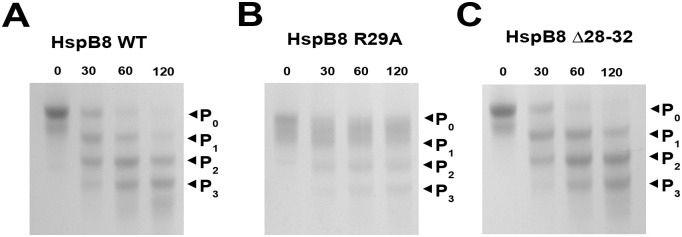
Urea gel-electrophoresis of HspB8 WT (A), and its R29A (B) and Δ28–32 (C) mutants in the course of phosphorylation by ERK1. Incubation time (min) is indicated above each lane. Arrows next to P_0,1,2,3_ represent bands of proteins carrying 0, 1, 2 or 3 phosphate groups, respectively.

Similar results were obtained upon phosphorylation of HspB8 by ERK1. In this case, both HspB8 WT and its Δ28–32 mutant were phosphorylated at multiple sites and therefore the prolonged phosphorylation resulted in the formation of three to four bands with higher electrophoretic mobility (Fig 6). Again, HspB8 WT and its Δ28–32 mutant were equally well phosphorylated by ERK1, whereas the R29A mutant was much less efficiently phosphorylated.

### Effect of mutations in the N-terminal domain of HspB8 on its chaperone-like activity

**Fig 7:**
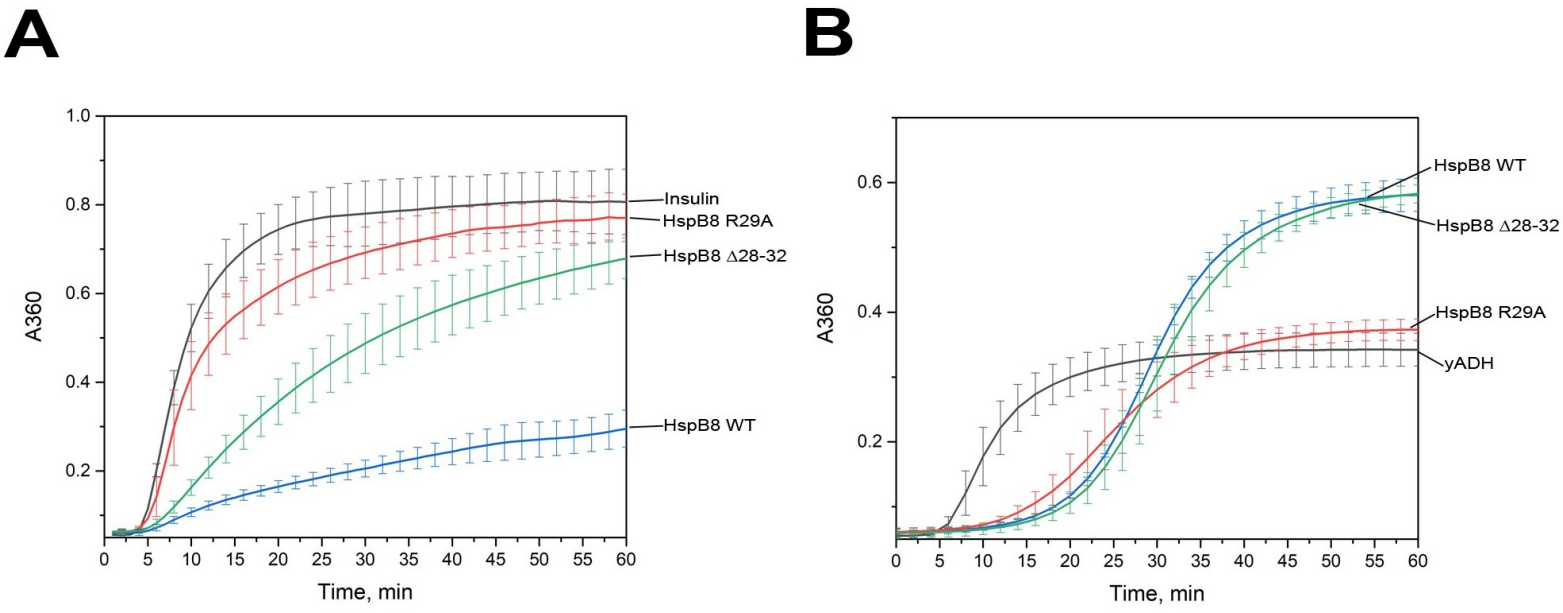
Chaperone-like activity of HspB8 WT and its mutants. A. Effect of HspB8 WT (blue), the R29A (red) and Δ28–32 (green) mutants on DTT-induced aggregation of insulin. B. Effect of HspB8 WT (blue), the R29A (red) and Δ28–32 (green) mutants on the heat–induced aggregation of yeast alcohol dehydrogenase (yADH). Data are representative of at least four independent experiments with error bars corresponding to standard deviation.

Two model protein substrates, namely insulin and alcohol dehydrogenase (yADH), were used for assessment of the chaperone-like activity of HspB8 and its two mutants. Reduction of disulfide bonds results in aggregation of the insulin B-chain (Fig 7A). HspB8 WT effectively prevented aggregation of insulin, whereas its Δ28–32 mutant was less effective and the R29A mutant was practically unable to prevent insulin aggregation.

Heat-induced aggregation of yADH was retarded by all HspB8 species, as judged from the presence of the pronounced lag phase (Fig 7B). However, in the case of HspB8 WT and its Δ28–32 mutant, long incubation was accompanied by a further increase of optical density that at the end of incubation was ~ 1.5 times higher than in the case of isolated yADH (Fig 7B). This was likely due to the interaction and co-precipitation of the WT protein and its Δ28–32 mutant (but not of the R29A mutant) with denatured yADH. Indeed, the pellet obtained after centrifugation of protein samples contained the WT protein and its Δ28–32 mutant and was completely free of R29A mutant.

## Discussion

**Fig 8:**
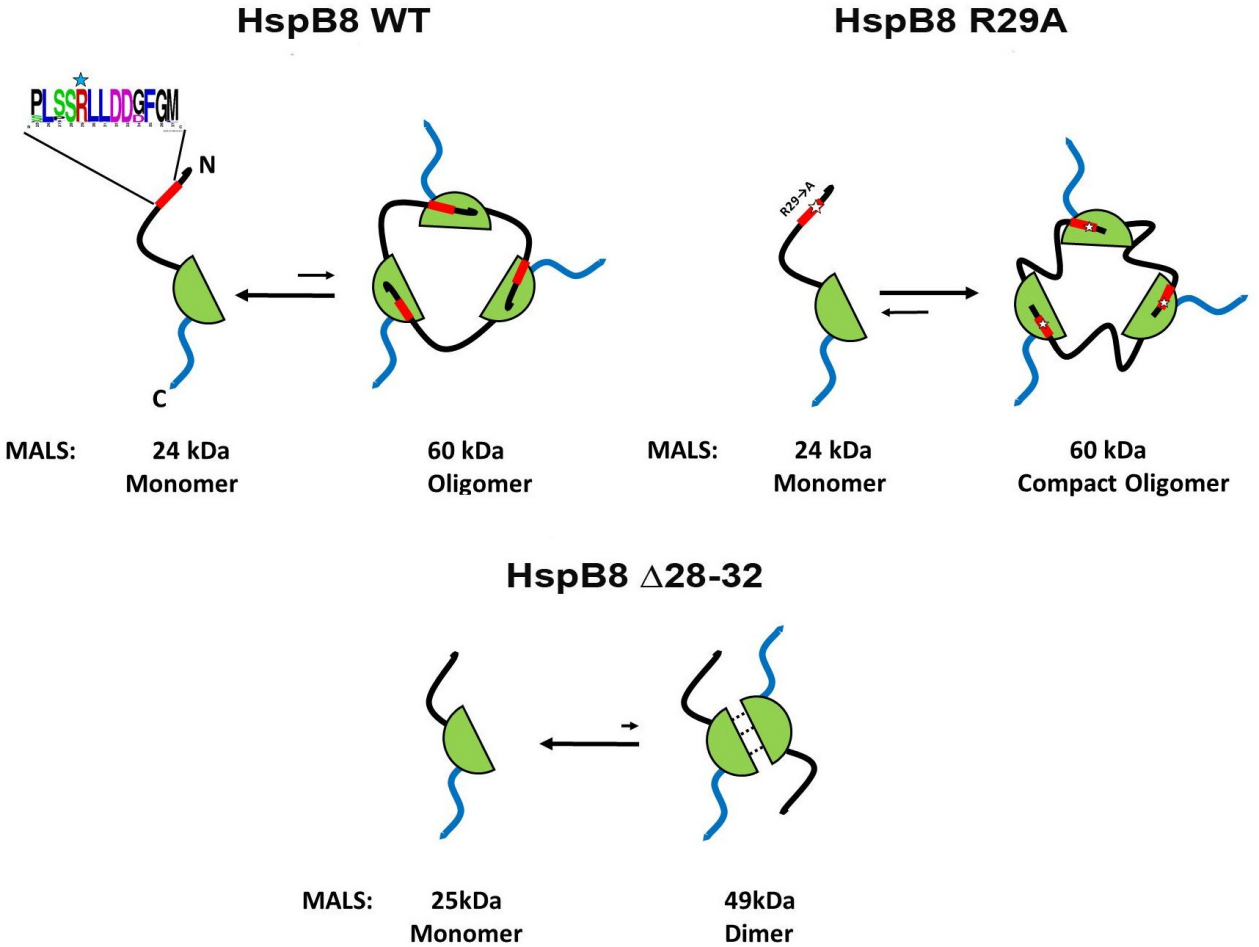
A scheme illustrating different oligomeric forms of HspB8 and its mutants. HspB8 WT is predominantly present as monomers with the small fraction of ~60 kDa oligomers (tentatively, tightly packed trimers or, less probable, unordered dimers). The R29A mutation promotes the formation of the 60-kDa oligomers, leaving only a small monomeric fraction. Oligomers with Mw of 60 kDa are tightly packed and their hydrophobic sites are predominantly masked. This decreases its interaction with bis-ANS, affects chaperone-like activity and decreases the probability of further oligomerization. The Δ mutant predominantly forms monomers with Mw of 24 kDa and a minor fraction of dimers with Mw of ~49 kDa.

For long the oligomeric structure of HspB8 has remained enigmatic. For instance, Chowdary et al., [33, 34] described HspB8 as a monomeric protein. Other biochemical data suggest that HspB8 can form homodimers, homotetramers or homooligomers [22, 35, 36]. By using SEC, we estimated the apparent Mw of HspB8 WT to be in the range of 33–36 kDa [20, 26, 37]. This value is different from calculated Mw of the HspB8 monomer (21.6 kDa) or dimer (43.2 kDa), which complicates unequivocal determination of the oligomeric state of HspB8. The utilization of SEC-MALS could help us to solve this question. By using this technique, we found that HspB8 WT is predominantly present in the form of monomers with Mw of 21–25 kDa (Figs 1, 8). HspB8 belongs to the group of intrinsically disordered proteins and likely forms an expanded conformation [32, 38]. In line with this, it eluted from the SEC column with an apparent Mw larger than that expected for the monomer. In addition to monomers, the HspB8 WT sample contains a small fraction of packed oligomers with the MALS-derived Mw of around 57–60 kDa (Figs 1, 8). The exact nature of this oligomer remains not fully clear. These oligomers are apparently in a slow equilibrium with monomers and, therefore, two well-separated peaks are detected on SEC profiles (Fig 1). The R29A mutant is predominantly present as oligomers with Mw of 59–60 kDa and contains only small quantities of monomer with Mw of 23–25 kDa (Figs 1, 8). This means that the R29A mutation substantially increases the fraction of the large oligomer. The Δ28–32 mutant of HspB8 was present as the monomer (22–24 kDa) and the dimer (46–48 kDa) and was unable to form 60-kDa oligomers. The oxidized Cys mutant of HspB8 containing replacements (C10S/C99S/C195S/N138C) formed an S-S crosslinked dimer with Mw of 45–46 kDa (Fig 1) and was unable to form large oligomers with Mw of ~60 kDa. Cys138 is located in the middle of the β7 strand in the interface of neighboring α-crystallin domains. Once β7 strands of α-crystallin domains of HspB8 pack antiparallel, forming the so-called APII type register [7, 39], the Cys138 residues become prone to form a disulfide bond fixing a nearly symmetric dimer with Mw of 45–46 kDa. Thus, the conservative pentapeptide ^28^SRLLD^32^ plays a crucial role in the formation of stable HspB8 oligomers with Mw of about 60 kDa. This oligomer seems to be very tightly packed and therefore it elutes on SEC later than more loosely packed dimers of the oxidized Cys mutant of HspB8 (Figs 1 and 8).

Different methods were used for characterization of physico-chemical properties of HspB8 species with mutations in the N-terminal domain. Both HspB8 WT and its Δ28–32 mutant bind bis-ANS thus demonstrating their hydrophobic properties. The R29A mutant, predominantly forming oligomers with Mw of about 60 kDa, also interacted with bis-ANS, however fluorescence of the complex formed by this protein and bis-ANS was lower than that of HspB8 WT or its Δ28–32 mutant (Fig 3). This means that the R29A mutant is less hydrophobic than two other HspB8 species. In the case of HspB8 WT and its Δ28–32 mutant, fluorescence of bound bis-ANS could be induced by excitation of Trp at 295 nm. This mechanism of excitation was less effective for the R29A mutant. Two Trp residues of HspB8 (Trp48 and Trp51) are located in the NTD. A decreased efficiency of energy transfer in the case of the R29A mutant can indicate that this mutation affects the orientation of the NTD of HspB8 and its location relative to bis-ANS binding sites.

As already mentioned, oligomers with Mw of ~60 kDa seem to be very tightly packed. This suggestion was indirectly confirmed by limited chymotrypsinolysis. Indeed, the rate of chymotrypsinolysis of HspB8 WT and its Δ28–32 mutant was much higher than that of the R29A mutant that remained intact even after very prolonged digestion (Fig 4).

HspB8 WT can be phosphorylated by cAMP-dependent and ERK1 protein kinases *in vitro*. Under these conditions, cAMP-dependent protein kinase predominantly phosphorylates Ser57 [40], whereas ERK1 phosphorylates multiple sites such as Ser24, Ser27 and Thr87 [41]. All these phosphorylation sites are located either in the NTD or on the border between the NTD and ACD. The R29A mutation strongly inhibited phosphorylation of HspB8 by both tested protein kinases (Figs 5 and 6). This indicates that replacement of the conservative Arg residue by Ala affects the structure of the NTD, provokes oligomerization and by this means inhibits phosphorylation of HspB8 by protein kinases. In this respect, it is worthwhile mentioning that the earlier published data [40, 41] indicate that phosphorylation, i.e. introduction of an additional negative charge to the NTD, increases the apparent Mw of HspB8. A similar effect was observed with the R29A mutant, where positively charged Arg was replaced by Ala. At the same time, the oligomeric structure of the Δ28–32 mutant of HspB8, lacking both Arg26 and Asp29 and having no extra charges, was similar to that of HspB8 WT.

The chaperone-like activity of HspB8 is usually lower than that of other sHsp members [30]. Indeed, HspB8 was ineffective in preventing heat-induced aggregation of ovotransferrin and S1 fragment of skeletal muscle myosin [20]. However, in good agreement with the earlier published data [30], HspB8 inhibited heat-induced aggregation of yADH and DTT-induced aggregation of insulin (Fig 7). Hence, the efficiency of chaperone-like activity depended on the nature of protein substrate. In the case of yADH, HspB8 WT and its Δ28–32 mutant inhibited only initial stages of substrate aggregation but provoked yADH aggregation on late stages of incubation (Fig 7B). The R29A mutant was relatively more effective in preventing yADH aggregation throughout the whole incubation (Fig 7B). An even more pronounced effect of the R29A mutation was observed with insulin as a model substrate. In this case, HspB8 WT demonstrated high chaperone-like activity. This activity was lower for the Δ28–32 mutant of HspB8 and was practically absent in the case of the R29A mutant (Fig 7A). Decreased chaperone-like activity of the R29A mutant can probably be explained by masking of its hydrophobic sites that are important for the interaction with protein substrates as a result of the amino acid substitution. Indeed, this mutant less effectively interacts with the hydrophobic probe bis-ANS (Fig 3).

The data presented mean that HspB8 is a very unusual small heat shock protein. In contrast to HspB1, HspB4 and HspB5 forming very large oligomers and HspB6 forming dimers, the predominant oligomeric form of HspB8 is a monomer. Under certain conditions and/or upon mutations in the NTD, HspB8 monomers can assemble into larger oligomers. The R29A mutation within the ^28^SRLLD^32^ pentapeptide leads to the formation of a hydrophobic patch consisting of Ala-Leu-Leu in the NTD of HspB8. HspB8 lacks the conservative I-X-I sequence in its C- or N-terminal domains thus leaving the hydrophobic β4/β8 groove of the ACD unoccupied. The newly formed Ala-Leu-Leu sequence of the R29A mutant can potentially interact with the β4/β8 groove and promote the formation of rather densely packed HspB8 oligomers. In addition, if this newly formed Ala-Leu-Leu tripeptide indeed interacts with the β4/β8 groove, the R29A mutation can affect the interaction of HspB8 with BAG3, the most prominent HspB8 partner [10, 42]. Interaction of BAG3 with HspB8 and its mutants is at present under investigation.

Under normal conditions, HspB8 WT is predominantly monomeric. This makes HspB8 a very valuable object for analyzing the properties and chaperone-like activity of monomeric sHsp. Earlier, the monomeric forms of the HspB1 homolog were obtained under rather harsh conditions leading to partial destabilization of the ACD [43, 44]. We suppose that even without any additional manipulations HspB8 WT can be used for functional characterization of monomeric forms of sHsp and their interaction with potential proteins substrates [45].

## Supporting information

S1 Raw images (PDF)
