# Supplementary material for: Replacement of Arg in the conserved N-terminal RLFDQxFG motif affects physico-chemical properties and chaperone-like activity of human small heat shock protein HspB8 (Hsp22)
Source: PLoS One. 2021 Jun 18;16(6):e0253432. doi: 10.1371/journal.pone.0253432 (PMC8213154; doi:10.1371/journal.pone.0253432)

Fig 4. Limited chymotrypsinolysis. SDS-PAGE 12,5%. Stain: Coomassie R250.
Mw marker: Pierce™ Unstained Protein MW Marker - 26610

Fig4A

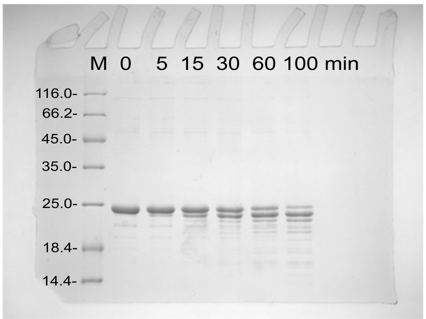

Fig4B

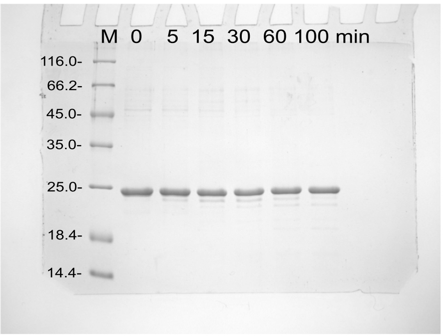

Fig4C

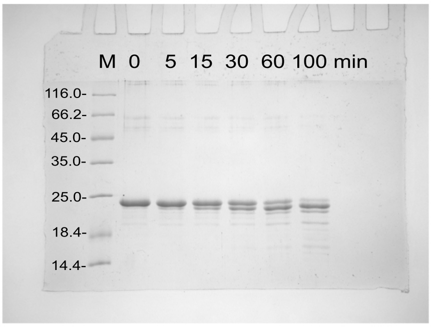

Fig 5. Phosphorylation of HspB8. Kinase PKA. UREA-PAGE 10%. Stain: Coomassie R250.

Fig5A Fig5B

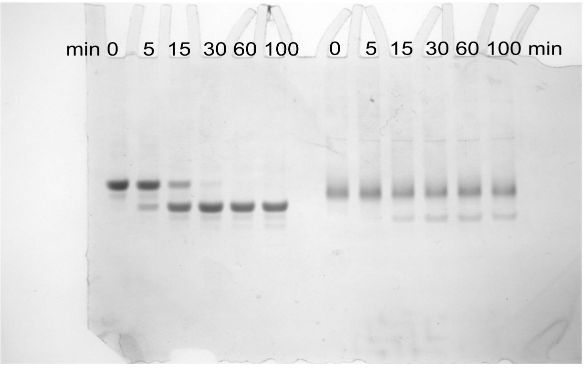

Fig5C

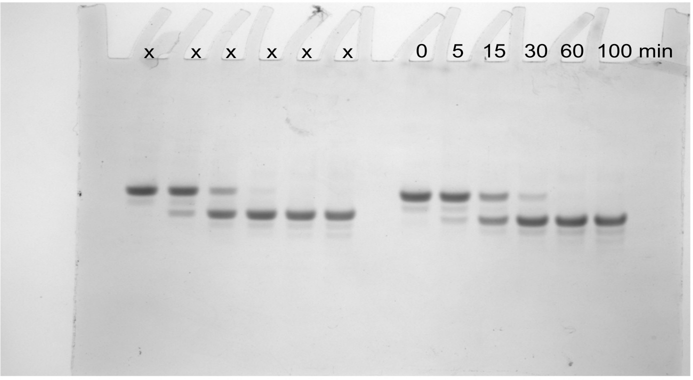

Fig 6. Phosphorylation of HspB8. Kinase ERK1. UREA-PAGE 10%. Stain: Coomassie R250.

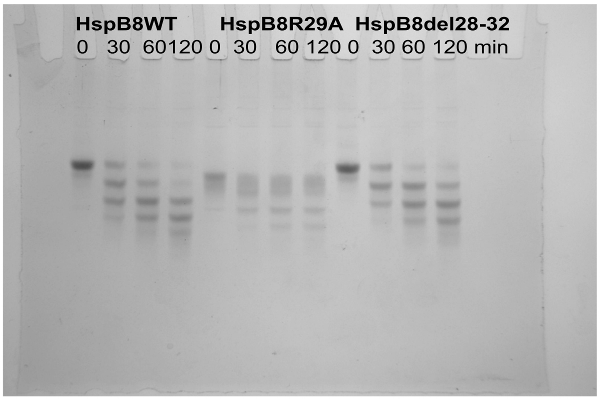

Supplement: S1 Raw images — (PDF) [file pone.0253432.s001.pdf]
